# A case of unusual histology of infantile lipoblastoma confirmed by *PLAG1* rearrangement

**DOI:** 10.1186/s40792-015-0042-4

**Published:** 2015-05-16

**Authors:** Toko Shinkai, Kouji Masumoto, Kentaro Ono, Eri Yano, Chie Kobayashi, Takashi Fukushima, Ryo Sumazaki, Kaishi Satomi, Masayuki Noguchi

**Affiliations:** Department of Pediatric Surgery, Faculty of Medicine, University of Tsukuba, Tsukuba, Japan; Department of Pediatrics, Faculty of Medicine, University of Tsukuba, Tsukuba, Japan; Department of Pathology, Faculty of Medicine, University of Tsukuba, Tsukuba, Japan

**Keywords:** Lipoblastoma, PLAG1, Immunohistochemistry (IHC), Fluorescence in situ hybridization (FISH)

## Abstract

Lipoblastoma, a relatively rare benign adipose neoplasm, predominantly affects children younger than 3 years of age. We herein report the case of a 7-month-old girl with an unusual myxomatous histology of lipoblastoma. A rapidly growing mass was detected in the subcutaneous area of the left buttock. Histologically, the tumor consisted of abundant myxoid stroma exhibiting cellular atypia and a high mitotic activity. Although the histological findings were unusual, the tumor was diagnosed as a lipoblastoma according to both PLAG1 immunohistochemistry and the presence of *PLAG1* rearrangement on fluorescence in situ hybridization.

## Background

Lipoblastoma is a specific and benign adipose neoplasm that consists of embryonic adipose tissue with the capacity for differentiation [1–3]. In general, lipoblastoma occurs most often in infancy and early childhood; nearly 90 % of cases are diagnosed before 3 years of age and 40 % are diagnosed in the first year of life [2, 3]. Adipose tumors are less common in children, accounting for only 6 % of pediatric soft tissue lesions. Lipoblastoma is a relatively rare tumor, estimated to comprise less than 19–30 % of all pediatric adipose masses [1, 2]. Lipoblastoma has an excellent prognosis, and complete local resection is required for treatment. Although this tumor is benign, with no potential for metastasis, it has a tendency to exhibit local recurrence [2, 4–6]. Recently, *PLAG1* rearrangement was identified to be a characteristic cytogenetic feature of lipoblastoma [7–14].

We experienced the case of a 7-month-old girl with a subcutaneous tumor demonstrating an unusual adipose and myxoid histology. As the tumor was suspected to be a lipoblastoma, we performed PLAG1 immunohistochemical staining (IHC) and an assessment for PLAG1 rearrangement using fluorescence in situ hybridization (FISH) to confirm the diagnosis. Positive results for the tumor led to the exact diagnosis.

## Case presentation

**Fig. 1:**
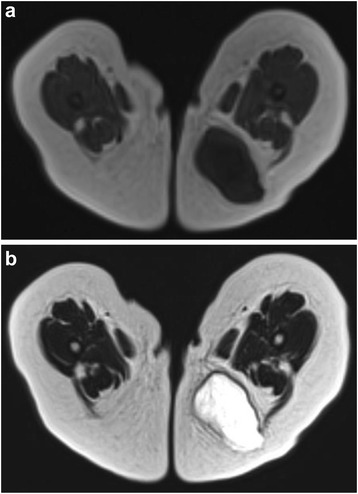
MRI images of the left buttock mass. **a** T1-weighted axial image showing a subcutaneous encapsulated mass measuring 4.6 × 2.9 × 3.9 cm. The mass was mostly homogenous with low-intensity signals. **b** T2-weighted axial images showing that the mass yielded mostly high-intensity signals

A 7-month-old previously healthy girl was admitted to our hospital for an investigation and treatment of a left buttock mass. The lesion was growing rapidly without tenderness. A physical examination showed a well-defined, firm, immovable, oval-shaped subcutaneous tumor measuring 4 cm in diameter. Laboratory data, including the levels of tumor markers, were within the normal ranges. Ultrasound sonography revealed a mass showing both uniform hypoechogenicity and small cystic lesions; the lesion did not include a hypervascular area. Magnetic resonance imaging (MRI) visualized a well-circumscribed subcutaneous mass measuring 4.6 × 2.9 × 3.9 cm in size that was hypointense on T1-weighted images and hyperintense on T2-weighted images (Fig. 1a,b), although it was not suppressed on fat-saturated T2-weighted images. A small portion of the mass was slightly enhanced on T1-weighted images with gadolinium enhancement. Therefore, the tumor was believed to primarily consist of myxomatous components with a small solid region. The preoperative diagnosis was a subcutaneous myxoid soft tissue tumor; there were no distant metastases. The patient underwent complete surgical resection of the mass on the fourth day after admission.

**Fig. 2:**
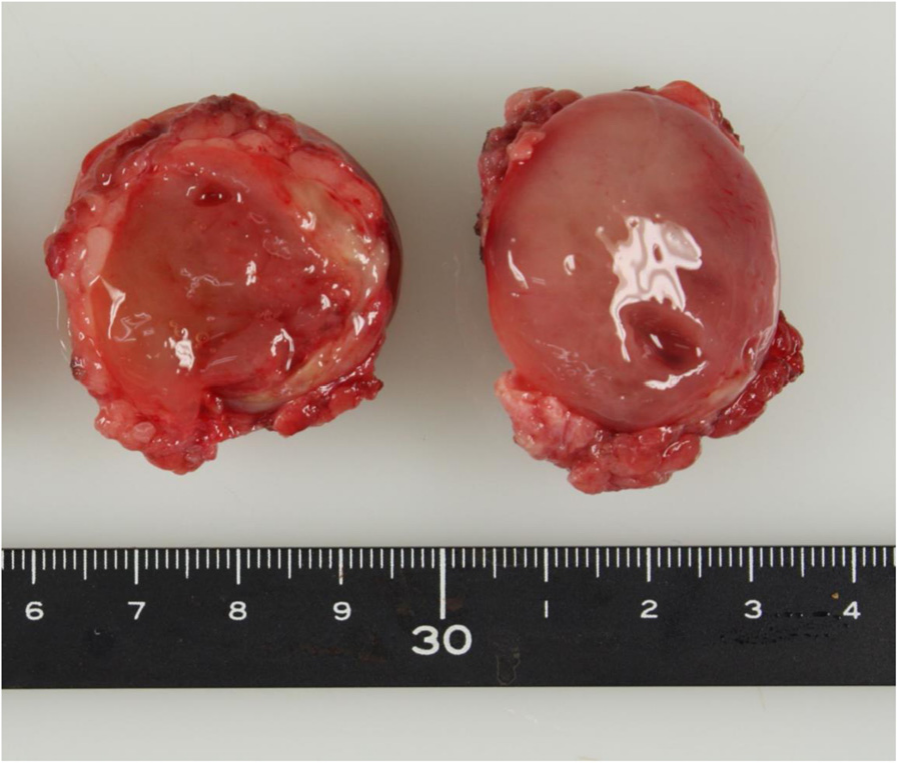
Macroscopic findings. The mass was well circumscribed with a glistening gelatinous cut surface and small cysts

**Fig. 3:**
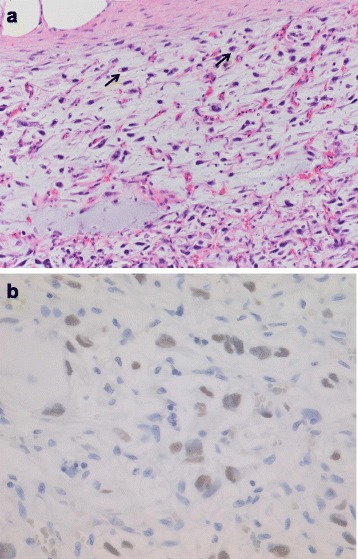
Histological findings. **a** Plexiform vascular networks and a prominently myxoid or mucoid matrix were observed in the tumor. The lesion was composed of the admixture of spindle and satellite cells with an atypical shape and hyperchromatic nuclei. The tumor did not contain mature adipose cells. Some lipoblast-like cells with small cytoplasmic vacuoles were observed (*arrows*, H&E × 200). **b** Immunohistochemical staining showed positivity for PLAG1 in many of the tumor cells (×400)

The resected tumor was found to be well encapsulated, measuring 4.5 × 4.0 × 4.0 cm in size. Macroscopically, the margin of the tumor was clear, and the content exhibited a predominantly myxomatous appearance (Fig. 2). Histologically, the lesion consisted of thin fibrous septa with a plexiform vasculature and prominent myxoid or mucoid matrix. Additionally, the tumor was composed of the admixture of spindle and satellite cells with an atypical shape and hyperchromatic nuclei, and some lipoblast-like cells with small cytoplasmic vacuoles were present (Fig. 3a). The mitotic count was 4 in 10 high-power fields, and the Ki-67 labeling index was 21.5 %. Although the tumor was suspected to be a lipoblastoma, the histological findings were unusual. Therefore, PLAG1 IHC and FISH for *PLAG1* rearrangement were performed. The results of PLAG1 IHC, which is specific for lipoblastoma, showed positivity in the tumor cells (Fig. 3b), and the investigation of the *PLAG1* rearrangement on FISH revealed positive findings (data not shown). Based on these results, we diagnosed the tumor as a lipoblastoma. The patient’s postoperative course was uneventful, and she has remained disease-free for 3 years.

## Discussion

Lipoblastoma is a benign lipomatous tumor that is usually detected in infancy. Indeed, approximately 90 % of these tumors are diagnosed in patients 0–3 years of age [1–4]. This lesion is slightly more predominant in males and occurs primarily in the extremities and trunk region, usually detected as a rapidly growing painless mass [1–6]. Although the prognosis of lipoblastoma is excellent due to its benign status with no potential for metastasis, wide and complete surgical resection is required for treatment. The rate of local recurrence varies between 9 % and 25 %, often attributed to incomplete resection, although recurrent tissue can be successfully re-excised [2, 4–6].

The imaging appearance of lipoblastoma depends on the proportion of fat relative to the amount of myxoid tissue. MRI is an important diagnostic tool for detecting lipoblastoma, and the features of this tumor often correspond to the pathological findings [4–6]. In general, MRI features can be summarized as including well-defined lobulated fatty masses with mostly high signal intensity on T1- and T2-weighted images [5, 6, 15, 16]. The fat suppression technique is helpful for confirming the existence of fatty components [5, 15]. The presence of fat is the predominant feature of lipoblastoma in many patients, particularly older children. In contrast, the detection of nonlipomatous myxoid components with only small elements of fat is a characteristic feature in infants [4, 5]. The myxoid components display enhanced contrast on MRI due to their rich capillary networks [4, 6].

Histologically, lipoblastoma is composed of small lobules containing mature and immature adipose tissue separated by connective tissue septa of varying thickness [1–3]. The adipocytes show a wide spectrum of maturation, with primitive stellate or spindled mesenchymal cells, multivacuolated lipoblasts, small signet ring lipoblasts, and mature adipocytes [1–4, 15, 16]. The observation of a myxoid stroma is more prominent in infants. Furthermore, a plexiform pattern of blood vessels is usually seen in the myxoid stroma in association with primitive mesenchymal cells [2, 15], and the tumor cells display the absence of significant nuclear atypia and pleomorphism with an extremely low mitotic rate [2, 3, 15]. Although an important differential diagnosis for lipoblastoma is myxoid liposarcoma, it is difficult to distinguish between these tumors due to the radiological and histological similarities of these lesions [1–4, 15, 16]. Lipoblastoma usually affects children younger than 10 years of age and predominantly those younger than 3 years of age. On the other hand, liposarcoma often occurs in the third through sixth decades of life. Liposarcoma is extraordinarily rare in patients below 10 years of age [2, 4, 6].

Given the age of our patient, a diagnosis of liposarcoma was unlikely. Furthermore, the histological findings were unusual for lipoblastoma, including the presence of large myxoid components, cellular atypia, and nuclear hyperchromasia with a high mitotic count. Therefore, it was necessary to further examine the tumor using immunohistochemistry and cytogenetic studies. Consequently, the tumor cells were found to be positive for PLAG1 IHC and demonstrated *PLAG1* rearrangement on FISH. Based on these results, we finally diagnosed the tumor as a lipoblastoma.

Cytogenetic karyotyping of lipoblastoma is characterized by the rearrangement of 8q11-13. *PLAG1* is a proto-oncogene located on chromosome 8q12 [7–14]. Approximately, 70 % of lipoblastomas exhibit *PLAG1* rearrangement located at 8q12, which can result in the transcriptional upregulation of this oncogene via promoter swapping [9, 10]. In addition, lipoblastoma demonstrates polysomy for chromosome 8 with or without *PLAG1* rearrangement [9, 10, 12, 13]. The detection of *PLAG1* rearrangement is used to distinguish lipoblastoma from other lipomatous tumors as well as liposarcoma [2, 3, 7, 8, 10, 13, 14].

Regarding the results of IHC for PLAG1 reported in a previous study [17], 8 of 10 cases of lipoblastoma were found to involve a positive expression on PLAG1 immunohistochemistry, whereas all 12 cases of liposarcoma displayed a negative PLAG1 expression. PLAG1 immunohistochemistry is also a very simple and useful diagnostic tool for distinguishing lipoblastoma from liposarcoma.

The sensitivity of *PLAG1* rearrangement has been reported 77 %, and the specificity of *PLAG1* rearrangement is 98 % [14]. Although FISH analysis of *PLAG1* rearrangement in lipoblastoma does not show high sensitivity, combined analysis of both IHC and cytogenetic study may increase the diagnostic accuracy of lipoblastoma with unusual histology.

## Conclusions

Lipoblastoma is a benign lipomatous tumor with an excellent prognosis. However, in some cases involving an atypical histology, analyses of the PLAG1 expression using both immunohistochemical and cytogenetic studies are needed to obtain an appropriate diagnosis.

## Consent

Written informed consent was obtained from the patient’s parents for publication of this case report and any accompanying images. A copy of the written consent is available for review by the Editor-in-Chief of this journal.
